# Arthroscopically assisted versus open reduction internal fixation for ankle fractures: a systematic review and meta-analysis

**DOI:** 10.1186/s13018-023-03597-9

**Published:** 2023-02-17

**Authors:** Guangming Zhang, Nong Chen, Linfeng Ji, Chengyi Sun, Sheng-Long Ding

**Affiliations:** 1 Department of Orthopaedic Surgery, Qingpu Branch of Zhongshan Hospital, Fudan University, 1158 Gong Yuan Dong Road, Qingpu District, Shanghai, 201700 China; 2 Fudan University, Fenglin Road, Xuhui District, Shanghai, 200030 China

**Keywords:** Ankle fracture, Arthroscopy, Open reduction internal fixation, Ankle fracture, Chondral lesion

## Abstract

**Background:**

Open reduction and internal fixation were routinely used to treat patients with unstable ankle fractures (ORIF). However, some patients may experience persistent ankle pain and disability following ORIF due to untreated intra-articular lesions. Moreover, ankle fractures may be treated with arthroscopically assisted reduction and internal fixation (ARIF). This study aimed to compare the feasibility and benefits of ARIF versus ORIF for ankle fractures.

**Methods:**

We performed this meta-analysis in accordance with the Preferred Reporting Items for Systematic Reviews and Meta-analyses (PRISMA) guidelines. A systematic search was conducted for comparative studies comparing ARIF and ORIF for ankle fractures. Nine studies were included in the analysis of clinical and secondary outcomes. In summary, we calculated the mean difference (MD), risk ratio (RR), confidence interval, and *p* value.

**Results:**

This meta-analysis demonstrated that the ARIF group achieved a higher Olerud–Molander Ankle (OMA) score (MD: 6.6; 95% CI 0.20 to 13.0; *p* = 0.04) and lower visual analog scale (VAS) score (MD: − 0.36; 95% CI − 0.64 to − 0.10; *p* = 0.01) at the final follow-up. Nevertheless, the smallest treatment effect of OMA score and VAS score did not exceed the minimum clinically important difference (MCID). There were longer surgery time (MD: 15.0; 95% CI 10.7 to 19.3; *p* < 0.01) and lower complication rates (RR: 0.53; 95% CI 0.31 to 0.89; *p* = 0.02) in ARIF compared with ORIF. The random-effect model suggested no significant difference in the arthritis change rate between the two groups.

**Conclusion:**

In summary, the results of this meta-analysis indicated that ARIF and ORIF are comparable in terms of providing pain relief and improving function for patients with ankle fractures. Therefore, the choice between the two techniques should be based on the patient's individual factors and the surgeon's personal preference.

## Introduction

Ankle fractures are among the most frequent injuries, averaging 169/100,000 times annually [1, 2]. Surgery is frequently used to treat unstable or dislocated ankle fractures to regain the ankle's stability and the articular surface's congruency. The most widely used method of treating unstable ankle fractures is generally acknowledged to be open reduction and internal fixation (ORIF) [3].

Even after the anatomical reduction, some patients still experience persistent ankle pain and disability, possibly as a result of untreated intra-articular lesions [4, 5]. Intra-articular disorders such as ligament disruptions or chondral lesions could be identified and treated in ankle fractures with arthroscopy [6]. According to a meta-analysis, chondral or osteochondral injuries were found in 65% of patients with ankle fractures [7]. For this reason, arthroscopically assisted reduction and internal fixation (ARIF) were proposed to treat ankle fractures. ARIF allowed verification of the anatomic reduction, examination of all intra-articular structures, and the immediate treatment of intra-articular lesions.

There has been a significant increase in ARIF utilization from 3.65 cases per 1000 ankle fractures in 2010 to 13.91 cases per 1000 ankle fractures in 2019 [8]. Recently, the optimal management for ankle fractures has regained popularity, and five new studies comparing ORIF versus ARIF were published [9–13]. Therefore, to obtain the most current and best scientific evidence, we conducted this meta-analysis to thoroughly compare the effectiveness of ORIF versus ARIF for ankle fractures.

## Methods

This meta-analysis was compiled according to the Preferred Reporting Items for Systematic Reviews and Meta-analyses (PRISMA) guidelines [14].

### Search strategy

We searched PubMed, Embase, and the Cochrane Library databases from inception to January 6, 2023. The following combination of keywords were used as the search strategy: (ankle OR distal fibular OR distal tibial OR malleolar) AND (arthroscopy OR arthroscopic OR arthroscopically). The search strategies are presented in Table 1. No language restriction was applied.

**Table 1:** Search strategy for PubMed and Ovid-Embase

Database	Search strategy	Search time	Number of identified study
PubMed	("ankle"[Title/Abstract] OR "distal fibula"[Title/Abstract] OR "distal tibia"[Title/Abstract] OR "bimalleolar"[Title/Abstract] OR "trimalleolar"[Title/Abstract] OR "malleolus"[Title/Abstract] OR "lateral malleolus"[Title/Abstract] OR "medial malleolus"[Title/Abstract]) AND "fracture" AND ("arthroscopy" OR "arthroscopic" OR "arthroscopically")	2023/1/6	363
Ovid-Embase	(ankle or distal fibula or distal tibia or bimalleolar or trimalleolar or malleolus or lateral malleolus or medial malleolus or ankle injur*).mp. and (fracture or fractures).mp. and (arthroscopy or arthroscopic or "arthroscopically").mp	2023/1/6	688

### Inclusion and exclusion criteria

Studies that met the inclusion criteria were included: (1) comparative studies comparing ARIF and ORIF; (2) studies with available full text; and (3) studies with a minimum of 6-month follow-up. The exclusion criteria were as follows: (1) case report; (2) case series; (3) review; (4) cadaveric studies; (5) animal studies.

### Study selection

Two independent authors conducted a literature search for relevant articles according to the search strategy above. Initially, the titles and abstracts were reviewed for all identified records. Thereafter, the full-text review was performed for potentially eligible studies. Any disagreements were resolved by consensus.

### Data extraction and quality assessment

Two reviewers independently extracted the following relevant information from included studies: country, the study design, population, fracture classification; age, sex, follow-up time, and outcome measures (OMs). The Cochrane Handbook for Systematic Reviews of Interventions 5.2.0. Higgins et al. [15] was to assess the methodological quality and risk of bias of randomized controlled trials (RCTs). The Newcastle–Ottawa scale (NOS) [16], a 9-pointed scale for selection, comparability, and outcome, was used to assess the methodologic quality of non-RCTs. Any discrepancies were resolved by discussion.

### Data analysis

All statistical analyses were performed using the software R (version 4.0.3) with the meta-package. Fixed-effects models were used without significant heterogeneity (*I*^2^ < 50%). Otherwise, random-effect models were adopted. The risk ratio (RR), along with a 95% confidence interval (CI), were calculated for dichotomous outcomes. The mean difference (MD) with a corresponding 95% CI was used to evaluate continuous outcomes. *p* < 0.05 was considered statistically significant. The minimum clinically important difference (MCID) refers to the smallest change in a health outcome measure that is considered to be of practical significance to patients [17]. The pooled results were compared with their MCID to identify clinical significance. Publication bias was investigated using a funnel plot and Egger’s test.

## Results

### Literature search

**Fig. 1:**
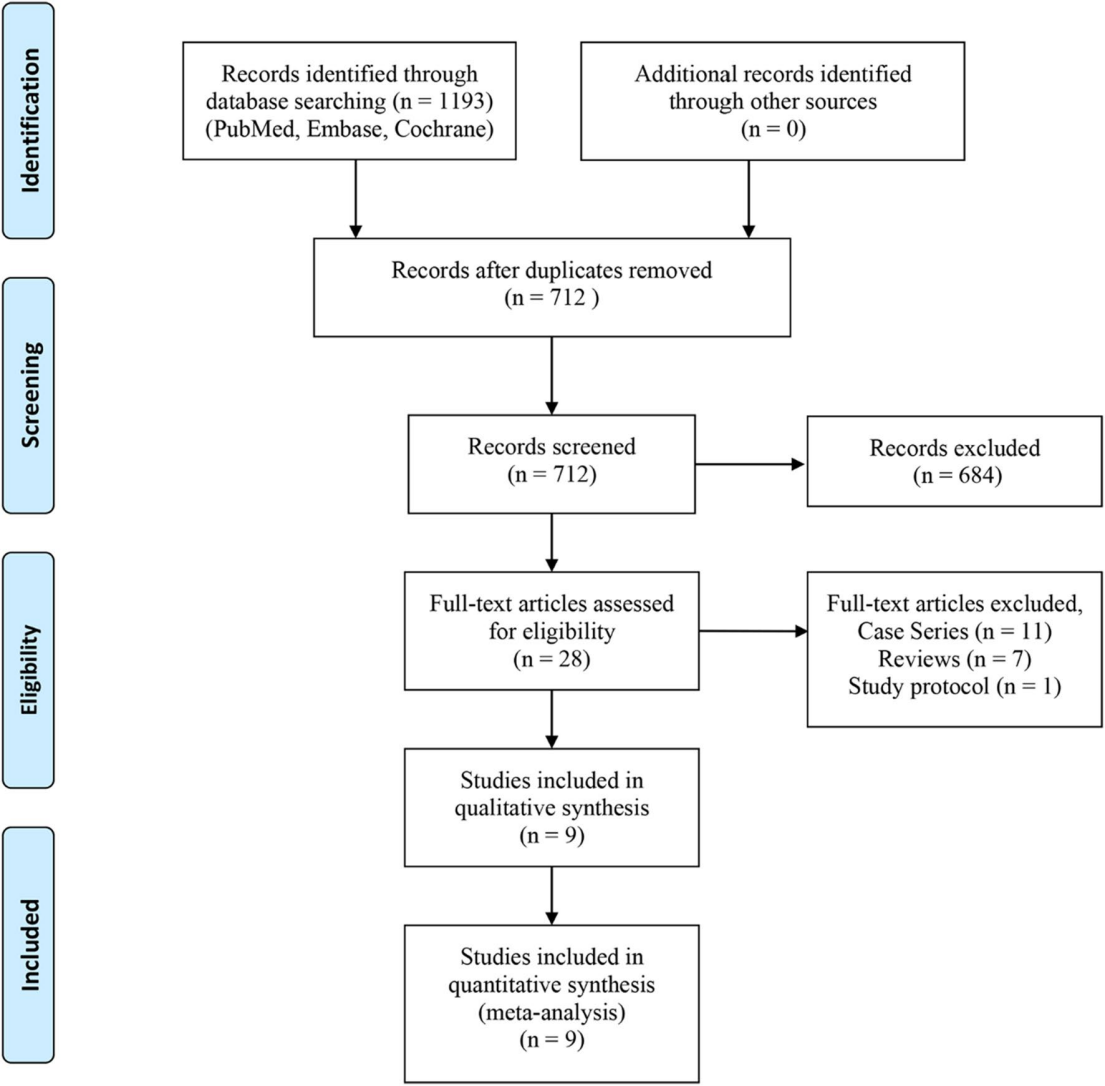
PRISMA study selection flow diagram. PRISMA, Preferred Reporting Items for Systematic Reviews and Meta-analyses

The initial literature search resulted in a total of 1193 studies. After duplicates were removed, 712 articles remained. After reviewing the titles and abstracts, 684 articles were excluded. Full text of the remaining 28 articles was assessed according to the eligibility criteria, in which nine studies were included in this meta-analysis [9–12, 18–21]. Figure 1 represents the flowchart indicating the progress of literature selection.

### Characteristics of included studies

**Fig. 2:**
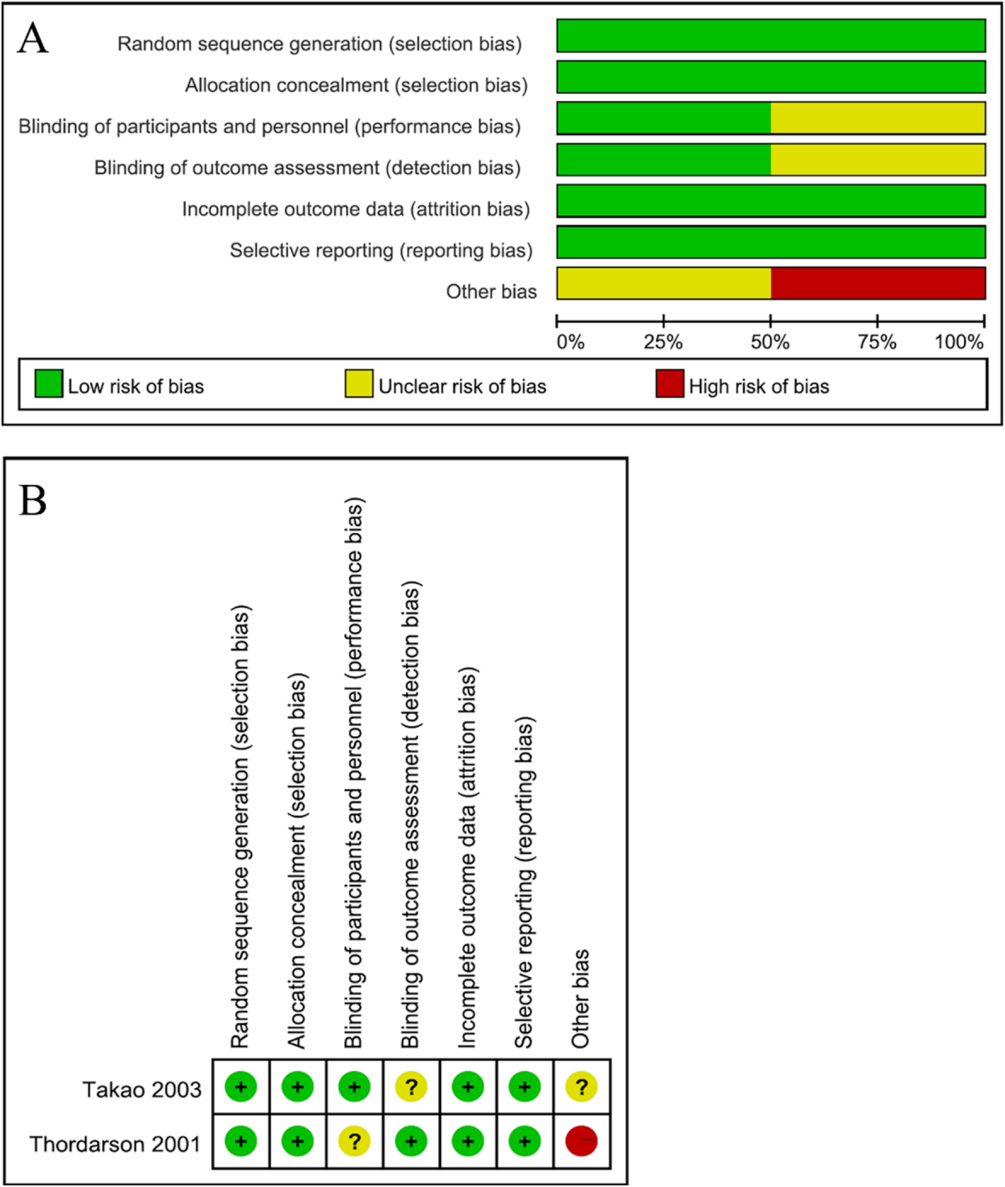
Quality assessment for randomized controlled trials (RCTs). **A** Risk of bias summary; **B** risk of bias graph

There were two randomized controlled studies (RCTs), six retrospective cohort studies, and a prospective cohort study. Two-hundred and seventy-three patients treated with ARIF and 306 patients treated with ORIF were compared in the nine studies that were included [9–13, 18–21]. The sample size in each study ranged from 19 to 113. The patients' ages ranged from 29 to 53 on average. Three ankle fracture classifications were documented in included studies, with four studies [10–12, 18] using the AO/OTA classification, three studies [9, 19, 20] adopting the Lauge–Hansen classification, and two studies [13, 21] utilizing the Herscovici classification (Table 2). NOS was used to appraise the methodologic quality of included studies. The average NOS score of non-RCTs was 6.1 points, and all of the ones that were chosen received 5–7 points, indicating that they were all of relatively high quality (Table 3). The methodological quality of RCTs is shown in Fig. 2.

**Table 2:** Characteristics of included studies

Author/year	Country	Design	No. of ARIF	No. of ORIF	Fracture classification, n	Mean age (years)	Sex (*M*/*F*)	Mean follow-up (months)	Outcome measures
Thordarson 2001	USA	RCT	9	10	SER, 16 PER,3	29	17/2	21	SF-36 AAOS
Takao 2003	Japan	RCT	41	31	SER,22 PAB, 50	ARIF: 36 ORIF: 38	48/24	ARIF: 40 ORIF: 41	AOFAS
Turhan 2012	Turkey	Retrospective	21	26	HS type B, 12 HS type C, 29 HS type D, 6	ARIF: 34 ORIF: 42	28/19	ARIF: 26 ORIF: 38	OMA VDC
Fuchs 2015	USA	Retrospective	24	27	AO/OTA type B, 33 AO/OTA type C, 18	ARIF: 38 ORIF: 40	31/20	67	OMA VAS
Angthong 2016	Thailand	Retrospective	16	29	SER, 33 PER, 12	ARIF: 48 ORIF: 45	24/21	10	QOR AC
Chiang 2019	China Taiwan	Retrospective	65	40	AO/OTA type B, 105	ARIF: 45 ORIF: 50	45/60	ARIF: 40 ORIF: 38	AOFAS VAS
Liu 2020	China	Prospective	34	43	HS type B, 41 HS type C, 36	ARIF: 37 ORIF: 38	48/29	ARIF: 60 ORIF: 62	OMA VAS
Baumbach 2021	Germany	Retrospective	25	25	AO/OTA type A, 2 AO/OTA type B, 39 AO/OTA type C, 9	ARIF: 46 ORIF: 53	21/29	ARIF: 53 ORIF: 48	OMA FAAM
Ceccarini 2021	Italy	Retrospective	38	75	AO/OTA type A, 42 AO/OTA type B, 71	38	53/82	38	ROM FAOS

RCT, randomized controlled trial; ARIF, arthroscopically assisted reduction and internal fixation; ORIF, open reduction and internal fixation; No., number; SER, supination-external rotation; PER, pronation-external rotation; PAB, pronation-abduction; HS, Herscovici classification; M, male; F, female; SF-36, the Medical Outcomes Study 36-Item Short Form Health Survey Physical Function scale; AAOS, American Academy of Orthopaedic Surgeons; AOFAS, American Orthopaedic Foot and Ankle Society; OMA, Olerud Molander Ankle score; VDC, van Dijk classification; QOR, quality of reduction; AC, arthritic changes; VAS, visual analog scale; FAAM, foot and ankle ability measure; ROM, range of motion; FAOS, Foot and Ankle Outcome Score

**Table 3:** Newcastle–Ottawa quality assessment of the included studies

References	Selection	Comparability	Exposure	Total score
Turhan et al.	2	1	3	6
Fuchs et al.	2	1	3	6
Angthong et al.	2	1	2	5
Chiang et al.	3	2	2	7
Liu et al.	3	2	2	7
Baumbach et al.	2	1	3	6
Ceccarini et al.	2	1	3	6

### Clinical outcomes

**Fig. 3:**
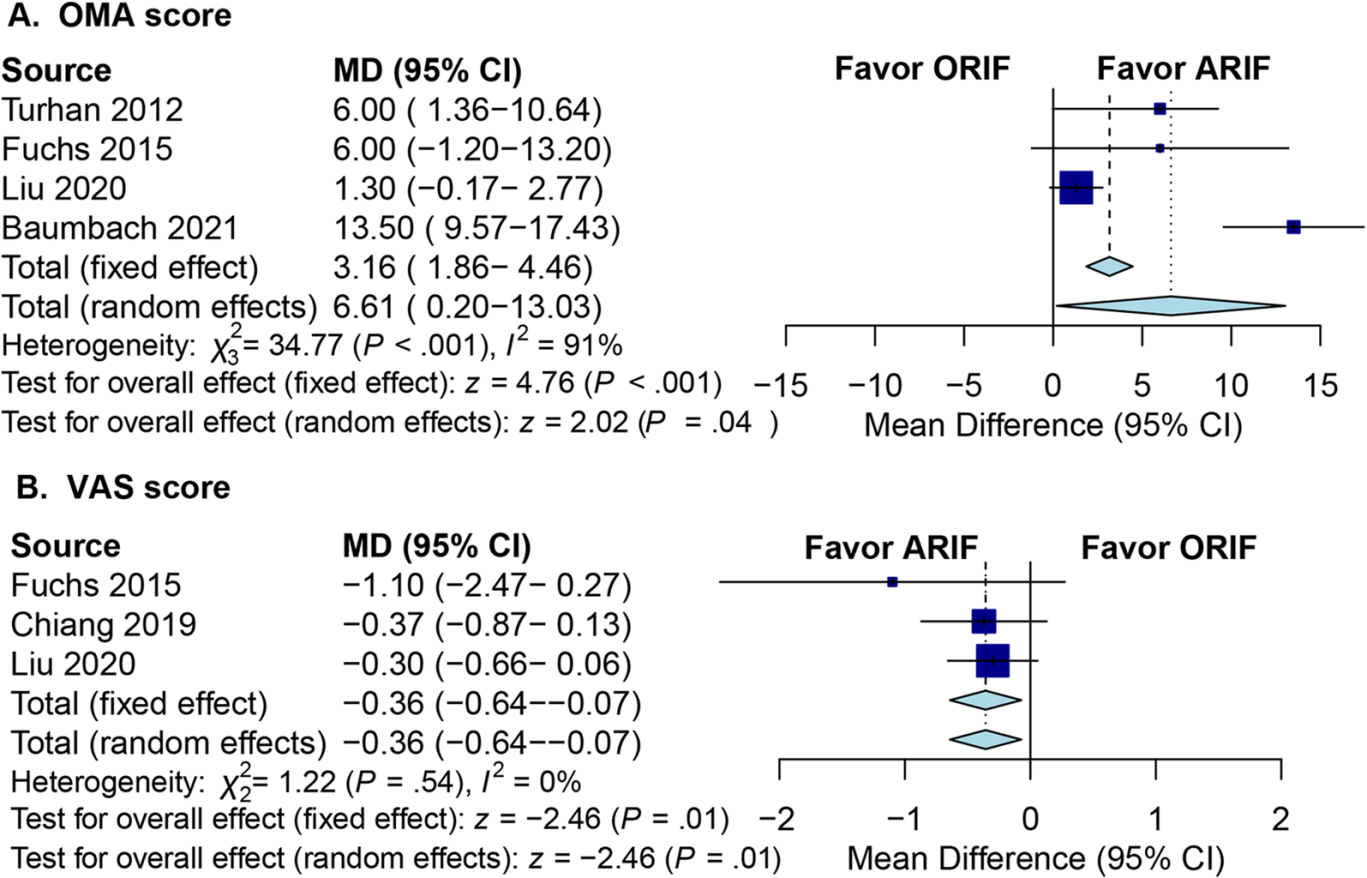
Forest plots of clinical outcomes for ARIF versus ORIF. **A** OMA score; **B** VAS score. (ARIF, arthroscopically assisted reduction and internal fixation; ORIF, open reduction and internal fixation; OMA, Olerud–Molander Ankle score; VAS, visual analog scale; MD, mean difference; CI, confidence interval.)

The American Orthopaedic Foot and Ankle Society (AOFAS) score, the Olerud–Molander Ankle (OMA) Score, or the Foot and Ankle Outcome Score are examples of functional outcomes of some studies, as shown in Table 4. The OMA score was a 100-point scale developed for clinical outcomes of patients with ankle fractures, with higher scores indicating better outcomes. Four studies involving 104 patients treated with ARIF and 121 patients treated with ORIF reported the OMA score at the final follow-up [10, 13, 18, 21]. In the random-effects model, we found a better outcome in the ARIF group measured with the OMA score (MD: 6.6; 95% CI 0.20 to 13.0; *I*^2^ = 91.0%; *Z* = 2.02; *p* = 0.04; Fig. 3A). The calculated MCID of OMA score in patients following ankle fractures determined to be between 10.5 and 15.0 [22]. However, the smallest treatment effect of OMA score (6.6) was below the MCID (10.5), suggesting that while the improvement was statistically significant, it may not have a significant clinical impact. Three studies were incorporated in the analysis of the visual analog scale (VAS) score at the final follow-up, and the pooled results from the fixed-effects model showed that ARIF was associated with less pain compared ORIF (MD: − 0.36; 95% CI − 0.64 to − 0.10; *I*^2^ = 0%; *Z* = − 2.46; *p* = 0.01; Fig. 3B). Zhang et al. determined the MCID for the VAS score to be 1.16 in a systematic review [23]. The pooled results in our meta-analysis showed that the smallest improvement seen in the VAS score (− 0.36) did not exceed the MCID (1.16), indicating that although the VAS score was statistically significant, it may not have a meaningful clinical impact.

**Table 4:** Functional outcomes and complications of included studies

Author/year	Functional OMs	Postoperative score (ARIF)	Postoperative score (ORIF)	Complications
Thordarson 2001	SF-36	97	96	NA
Takao 2003	AOFAS	91	87.6	ARIF: no complication ORIF: two superficial wound infections
Turhan 2012	OMA	92.3	86.3	ARIF: no complications ORIF: three wound complications
Fuchs 2015	OMA	90	84	ARIF: one nerve injury, one wound complication, and three residual numbness ORIF: four residual numbness
Angthong 2016	NA	NA	NA	ARIF: one wound complication ORIF: one loss of reduction
Chiang 2019	AOFAS	92.3	89.8	ARIF: one superficial peroneal paresthesia, one wound complication, and three hardware irritation ORIF: three superficial peroneal paresthesia, one nonunion, five wound complications, and two hardware irritation
Liu 2020	OMA	97.9	96.6	ARIF: two superficial peroneal paresthesia, one hardware irritation ORIF: three wound complications, one infection, and four hardware irritation
Baumbach 2021	OMA	90	75	ARIF: three major complications ORIF: two major complications
Ceccarini 2021	FAOS	86	82	No major complications

OMs, outcome measures; SF-36, the Medical Outcomes Study 36-Item Short Form Health Survey Physical Function scale; AOFAS, American Orthopaedic Foot and Ankle Society; OMA, Olerud Molander Ankle score; NA, not available; FAOS, Foot and Ankle Outcome Score; ARIF, arthroscopically assisted reduction and internal fixation; ORIF, open reduction and internal fixation

### Secondary outcomes

**Fig. 4:**
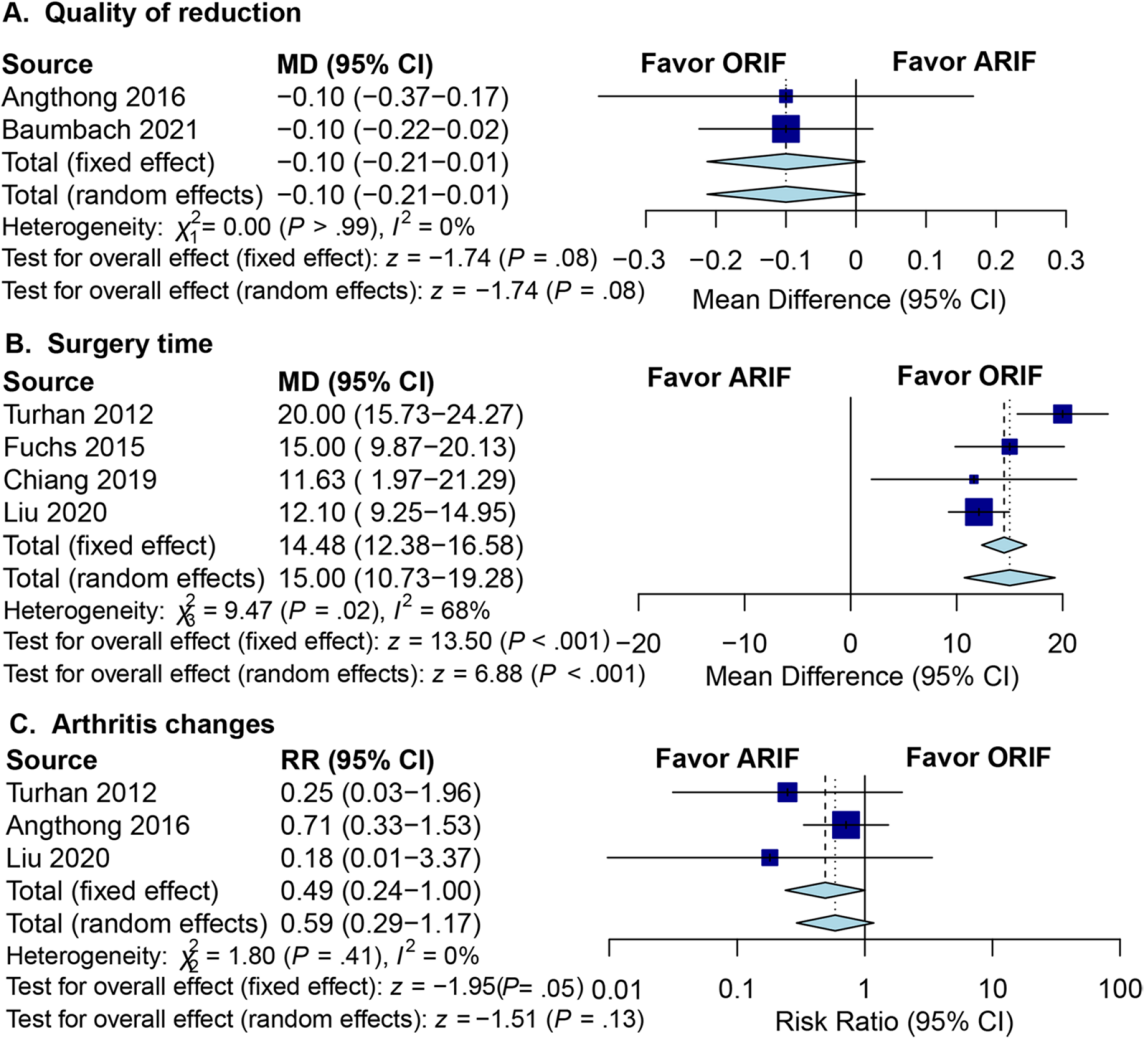
Forest plots of secondary outcomes for ARIF versus ORIF. **A** Quality of reduction; **B** surgery time; **C** arthritis changes. (ARIF, arthroscopically assisted reduction and internal fixation; ORIF, open reduction and internal fixation; MD, mean difference; RR, risk ratio; CI, confidence interval.)

The quality of reduction was assessed for each fracture separately (< 2 mm = 1 point; ≥ 2 mm = 2 points for dislocation/gap in any plane), and the average point was calculated for each patient [24]. Angthong et al. and Baumbach et al. assessed the quality of reduction after ARIF and ORIF for ankle fractures [9, 10]. According to the pooled results, there was no significant difference between the ARIF and ORIF groups concerning the quality of reduction (Fig. 4A).

Four studies reported the surgery time. The results showed that the heterogeneity test for the surgery time was statistically significant. Hence the random-effects model was used [12, 13, 18, 21]. The pooled results revealed that the ARIF group had a longer surgery time than the ORIF group (MD: 15.0; 95% CI 10.7 to 19.3; *I*^2^ = 68.0%; *Z* = 6.88; *p* < 0.01; Fig. 4B). Arthritis changes in the ankle joints were assessed according to van Dijk classification system at the final follow-up [25]. Three studies documented the arthritis changes rate, in which 71 patients were treated with ARIF and 88 patients were treated with ORIF [9, 13, 21]. The pooled analysis from the random-effects model suggested that there was no significant difference in the arthritis changes rate between the two groups (Fig. 4C).

**Fig. 5:**
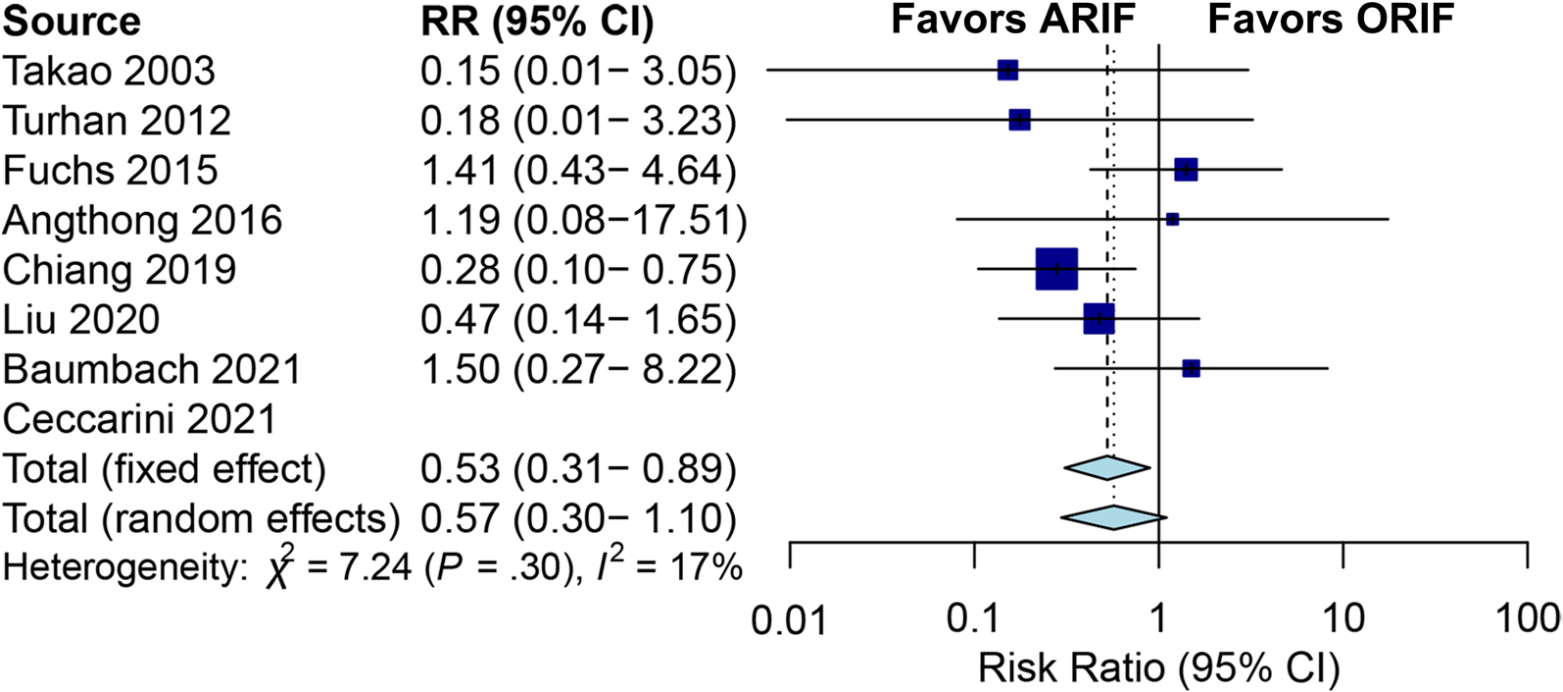
Forest plots of complication rates for ARIF versus ORIF. (ARIF, arthroscopically assisted reduction and internal fixation; ORIF, open reduction and internal fixation; RR, risk ratio; CI, confidence interval.)

**Fig. 6:**
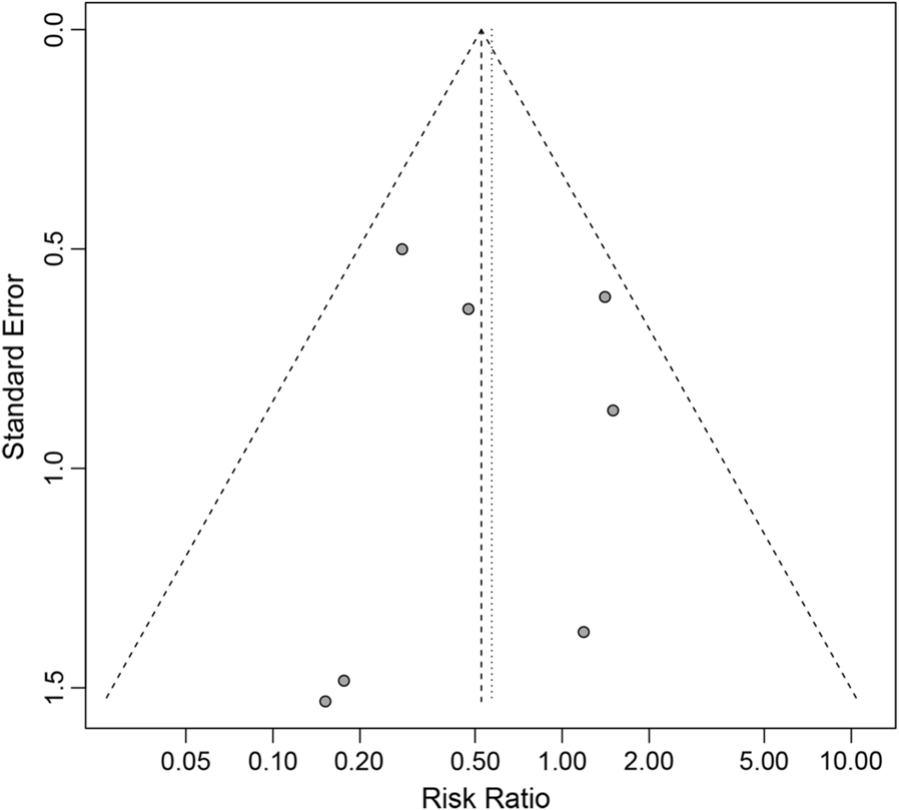
Funnel plot of eight included studies for complication rates

Complications of included studies, such as superficial peroneal paresthesia, hardware irritation, or wound complications, are presented in Table 4. Eight studies reported complication rates were included, involving 264 patients treated with ARIF and 286 patients treated with ORIF [9–13, 18, 19, 21]. The pooled RR using the fixed-effects model for complication rates was 0.53, demonstrating that more complications in the ORIF group (RR: 0.53; 95% CI 0.31 to 0.89; *I*^2^ = 17.1%; *Z* = − 2.37 *p* = 0.02; Fig. 5). The funnel plot showing the symmetric distribution of the dots and Egger’s test (*p* = 0.98) suggested that there was no publication bias (Fig. 6).

## Discussion

This meta-analysis sought to comprehensively assess the efficiency of ARIF versus ORIF for ankle fractures. The pooled results of nine included studies showed statistically significant higher OMA score, lower VAS score, longer surgery time, and lower complication rates in ARIF compared with ORIF for patients with ankle fractures. Nevertheless, no significant difference was found in arthritis changes and the quality of reduction between the ARIF and ORIF groups. Additionally, neither the OMA score nor the VAS score showed improvements that met the minimum level considered to be clinically significant. As a result, these scores were not deemed to have a meaningful clinical impact. To sum up, this meta-analysis found that there was no difference in the effectiveness of ARIF and ORIF in terms of providing pain relief and improving function for patients with ankle fractures.

ARIF for ankle fractures is an acceptable treatment option for ankle fractures involving chondral or osteochondral injuries. However, ARIF for patients with ankle fractures is much debated, and there is no consensus on the optimal treatment for ankle fractures [11]. Besides, ankle arthroscopy for reducing ankle fractures requires a higher cost and takes longer surgical time.

Although ankle arthroscopy has been proven to be effective in the treatment of osteochondral lesions of the talus [26], ARIF treatment for patients with ankle fractures is still debated, and there is no consensus on the treatment. Chiang et al. compared ARIF and ORIF for ankle fractures in a retrospective study and reported proven lower incidences of complications in the ARIF group [12]. Nevertheless, Fuchs et al. reported no significant difference on the clinical outcomes of patients with ankle fractures who underwent ankle ORIF and ARIF [18]. A meta-analysis of four studies was conducted in favor of ARIF for ankle fractures, but there were some limitations to be addressed [27]. Firstly, there were not enough included studies for generalization to be possible. Secondly, the included four studies reported different functional outcomes, and the meta-analysis pooled these outcome measures, which involved a high heterogeneity. Thirdly, due to limited data in the included studies, radiological outcomes, complication rates, and surgery time were not pooled in the former meta-analysis. Therefore, this present meta-analysis was conducted to determine the effectiveness of ARIF versus ORIF for ankle fractures.

In this meta-analysis, we used the MCID as a reference to determine the clinical significance of our findings. The MCID represents the smallest change in a patient's condition or health outcome that is considered to be meaningful and important from a clinical perspective [17]. It is often used as a benchmark for evaluating the effectiveness of a medical treatment or intervention, as well as for determining whether a change in a patient's condition is significant enough to warrant a change in treatment [22]. By comparing the pooled results with the MCID, we aimed to gain a clearer understanding of the clinical significance of our findings. OMA score and VAS score at the final follow-up were our clinical outcomes. OMA score is a validated clinical scoring system used to evaluate the severity of ankle fractures [28]. Penning et al. conducted a cohort study to determine the MCID of the OMA score for patients with ankle fractures that underwent surgeries, reporting that the calculated MCID for OMA score ranges from 10.5 to 15.0 [22]. Ankle arthroscopy was not found to improve OMA score in a retrospective study by Fuchs et al. that compared ankle ORIF with and without ankle arthroscopy [18]. Liu et al. compared arthroscopic reduction percutaneous fixation and ORIF for ankle fractures, showing no significant difference in OMA score at the final follow-up [13]. Baumbach et al. reported an OMA score in ARIF that was significantly higher, which might be explained by the early recognition and management of intra-articular lesions using arthroscopy [10]. This meta-analysis pooled four studies and found a statistically significant higher OMA score in the ARIF group. However, the smallest treatment effect of OMA score (6.6) did not exceed the MCID (10.5), indicating that the improvement of OMA score was not clinically significant. The VAS score was to quantitatively evaluate the pain intensity, with lower scores indicating less pain [29]. The pooled MD in the VAS score (− 0.36) was also below the MCID (1.16), suggesting that the improvement in pain intensity was not deemed to be clinically significant [23].

The quality of reduction for ankle fractures was the most significant variable affecting the clinical result [3, 24]. There was no difference between the ARIF and ORIF groups, according to this analysis of two studies [9, 10]. Liu et al. reported that three patients (7%) had osteoarthritis changes which were described as grade 1 according to the van Dijk classification system in the ORIF group. And no such changes in the ARIF group [13]. Turhan et al. found that only one patient (5%) had Grade 1 osteoarthritis change in the ARIF group [21]. However, three patients had Grade 1, and two patients had Grade 2 osteoarthritic change in the ORIF group. This meta-analysis pooled three studies and demonstrated no significant difference in osteoarthritic change rate between the two groups in the random-effect model. Stukens et al. demonstrated that the presence of early cartilage damage after an ankle fracture is a reliable indicator of the onset of osteoarthritis [6]. Occult cartilage lesions could be detected and treated using arthroscopy in the ARIF group, but the ARIF was not found to be superior than ORIF in osteoarthritis changes after surgery for ankle fractures. Further, RCTs with long-term follow-up were needed to identify the difference between ARIF and ORIF groups.

To the best of our knowledge, this is the first meta-analysis which compares the efficacy of ARIF versus ORIF for ankle fractures, taking into account six outcome measures and nine studies. There were several limitations to this study. First, different types of ankle fractures were included, with high heterogeneity. Second, non-RCTs were included, and a larger sample RCTs are needed to further to verify the results of this meta-analysis. Several studies suggested that the compartment pressure increased within the ankle joint during the ARIF procedure, which may lead to dreadful outcomes [21, 30]. Nevertheless, this meta-analysis included nine studies and found no compartment syndrome secondary to fluid extravasation in the ARIF group. Besides, this study found a lower complication rate in the ARIF group, which indicated that ARIF technique is relatively safe.

In conclusion, ARIF was not found to be superior to ORIF in pain relief and function improvement for patients with ankle fractures. Ultimately, the choice between ARIF and ORIF will depend on the specific case and the surgeon's assessment of the patient's needs and goals.

## Data Availability

Data under study are available on request from the corresponding author, which is not publicly available due to privacy or ethical restrictions.

## Abbreviations

ARIF: Arthroscopically assisted reduction and internal fixation
ORIF: Open reduction and internal fixation
AAOS: American Academy of Orthopaedic Surgeons
VAS: Visual analog scale
OMA: Olerud Molander Ankle score
FAAM: Foot and ankle ability measure
FAOS: Foot and ankle outcome score
SF-36: The Medical Outcomes Study 36-Item Short Form Health Survey Physical Function scale
AOFAS: American Orthopaedic Foot and Ankle Society
SER: Supination-external rotation
PER: Pronation-external rotation
PAB: Pronation-abduction
